# Antioxidant, Antifungal, Antibiofilm, and Cytotoxic Activities of *Mentha* spp. Essential Oils

**DOI:** 10.3390/medicines5040112

**Published:** 2018-10-21

**Authors:** Annarita Stringaro, Marisa Colone, Letizia Angiolella

**Affiliations:** 1 National Center for Drug Research and Evaluation, Italian National Institute of Health, Viale Regina Elena, 299, 00161 Rome, Italy; annarita.stringaro@iss.it (A.S.); marisa.colone@iss.it (M.C.); 2 Department of Public Health and Infectious Diseases, Sapienza University of Rome, P.le Aldo Moro, 5, 00185 Rome, Italy

**Keywords:** essential oil, *Mentha* spp., antioxidant, antifungal, antibiofilm, toxicity

## Abstract

Since ancient times, plants have been used to preserve food, or for their health properties. Essential oils are complex mixtures of volatile compounds that are obtained from botanical material, specifically from aromatic plants. Lamiaceae is one of the most important families in the production of essential oils, as it has both antioxidant and antimicrobial properties. The essential oils of *Mentha* (the Lamiaceae family) have been extensively studied for their biological actions. In this review, we report the antioxidant, antifungal, antibiofilm, and cytotoxic properties of *Mentha* spp. essential oils. The first objective is to provide comprehensive information about the use of essential oils in the treatment of fungal infections, or as antioxidants and integrative anticancer therapy. The second is to explore the evidence supporting its effectiveness in treating diseases without causing any serious adverse reactions.

## 1. Introduction

Plant essential oils (EOs) are produced predominantly using steam distillation, but can also be generated using fermentation, crushing, extraction, hydrolysis, and airing [1]. EOs are used extensively in cosmetics in many different aspects as perfumes, in antiseptic applications, and in domestic cleaning products [2,3]. They are volatile liquids or semi-liquids [4] that are limpid, but are rarely colored, and are soluble in organic solvents. All of the organs of the plants can synthesize EOs, which are stored in secretory compartments as cavities, canals, epidermic cells, or glandular trichomes. EOs are complex mixtures of terpenoides containing sesquiterpene and monoterpene, and their oxygenated derivatives. EOs may also incorporate a variety of other molecules such as fatty acids, oxides, and sulfur derivatives [5]. Both the terpenoid and phenylpropanoid families, which are sometimes identified as the principal constituents of several EOs, can constitute 85% of the total concentration of the oil. There are about 3000 well-recognized EOs, of which 300 are widely sold [6]. Various factors influence the chemical compositions of EOs, such as their geographic location, the seasonal period in which they are collected, the soil composition and cultivation method, their storage, and the oil extraction method [7,8]. The high level of interest in research regarding EOs is due to their many biological and medical properties [9]. They are generally recognized as safe, and they can act synergistically with other compounds, which are promising factors for their use as bioactive compounds [10].

Based on the number of search results in the PubMed database, the published studies on their antimicrobial, antioxidant, and anti-tumoral activities are 2671, 1186, and 108, respectively [11].

### Mentha spp. Essential Oils

Many aromatic plants used in medicine, food, and pharmaceutical industries belong to the Lamiaceae family. In this family, *Mentha* is a well-known genus that includes 25–30 species that are generally grown in temperate areas around the world, particularly in Europe, North America, North Africa, Asia Minor, the northern parts of Iran, and near the east (Syria, Ethiopia). *Mentha* spp. includes plants that exhibit important biological activities and have high morphological variability and a great chemical diversity with respect to their EOs [10]. 

**Figure 1:**
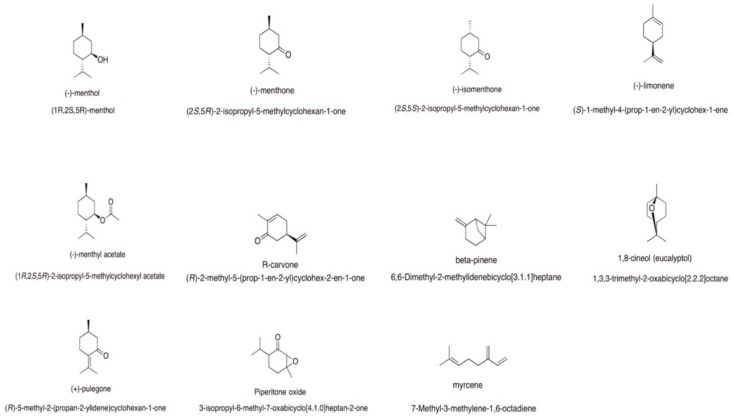
Chemical structures of the main components of *Mentha* spp. essential oils (EOs).

For this reason, *Mentha*-derived EOs have been used as a folk remedy for respiratory diseases such as bronchitis, sinusitis, tuberculosis, and the common cold [12]. *Mentha* acts as a good expectorant. The chemistry of *Mentha* EOs is complex and high variable. The main constituents of the most commonly used *Mentha* EOs revealed by gas chromatography–mass spectrometry (GC-MS) analysis show the presence of menthol, menthone, limonene, isomenthone, menthyl acetate, carvone, β-pinene, 1,8-cineole, pulegone, piperitone oxide, and micene. Each species has a characteristic prevalent compound (Figure 1). Studies that have already been carried out with *Mentha* spp. have shown antimicrobial activity related to some species of this genus. The most cited activities of the plant are its antiviral, antibacterial, antifungal [13], high antioxidant, and cytotoxic properties [14], and also other properties such as its antinociceptive, anti-inflammatory, and antiallergic qualities [15]. This review will focus on some of these properties.

## 2. Antioxidant Properties 

In recent years, there has been an increasing interest in the consumption of EOs as natural antioxidants [16]. It is well-known that reactive oxygen species (ROS) cause damage to cellular macromolecules, and they are implicated in the development of many human diseases. Under many pathological conditions the oxidation–reduction (redox) potential imbalance cannot remove excessive amounts of ROS [17]. Oxidant species such as hydrogen peroxide (H_2_O_2_) and superoxide (O_2_^−^) are produce following the phagocytosis of the pathogen by these cells as part of their machinery to respond to harmful insults [18]. Excessive nitric oxide (NO) production and increased levels of prooxidant species may lead to damage and poor perfusion of the vital organs of the host, contributing to multiple organ failure; thus, to counteract this response, antioxidant pathways are activated [19]. Free radicals generated by damaged membranes, when combined with EOs, produce radicals with scavenging activity. 

Natural antioxidants such as phenolic compounds can be found in many plants. The antioxidant activity of essential oils does not always depend on its main component but can be modulated by other components [20]. 

Antioxidant activity can be evaluated using various methods, and analytical tools are utilized for measuring antioxidant content and total antioxidant capacity evaluation. The methods of antioxidant capacity evaluation include counting spectrometry, chromatography, and electrochemical techniques [21]. Generally, the most frequently used methods are 2,2-diphenyl-1-picrylhydrazyl, (DPPH), 2,2’-azino-bis (3-ethylbenzthiazoline-6-sulphonic acid), (ABTS), and others [22]. 

The main reaction occurring in spectrometric techniques is between a radical, radical cation, or a complex and an antioxidant molecule donor of a hydrogen atom. DPPH is a stable free radical, and the assay is based on electron transfer that produces a purple solution in ethanol, with an absorption band with a maximum of around 520 nm. When DPPH reacts with a hydrogen donor, it generates the reduced molecular form (DPPH), and the purple color disappears. The antioxidant concentration is linearly correlated with the absorbance diminution. The standard antioxidant used is Trolox. The standard curve was linear between 25–800 mM Trolox [23].

The biamperometric antioxidant capacity assay also uses the redox couple ABTS+/ABTS. The ABTS cation radical (ABTS+) [24], which absorbs at 743 nm (giving a green color), is formed by the loss of an electron by the nitrogen atom of ABTS. In the presence of Trolox (or another hydrogen-donating antioxidant), the nitrogen atom quenches the hydrogen atom, causing the solution’s decolorization. ABTS can be oxidized by potassium persulfate [24,25] or manganese dioxide [26]. The standard curve was linear between 25–600 µM of Trolox. Unfortunately, the evaluation of the antioxidant activity of EOs remains a critical issue, because many “tests” are unsuitable and provide contradictory results [27].

*Mentha piperita* L. is a plant native to the Mediterranean region that is popularly known as peppermint; it is used medicinally for its antiproliferative and antioxidant actions. The plant is also used worldwide, especially in the perfume and food industries, for its taste and fragrance. 

*Mentha piperita* EO possesses antiradical activity with respect to DPPH and hydroxyl (OH^−^) radicals; indeed, Schmidt et al. [28] have reported the antiradical activity of this EO for DPPH as (IC_50_), while some authors have reported its radical scavenger activity against the ABTS radical [29]. 

Recently, similar results were confirmed by Sun et al. [30], specifically the action of peppermint EO as a scavenger of hydroxyl radicals, and the potential for it to be an antioxidant at concentrations ≥200 μg/mL. da Silva Ramos et al. [31] reported an antioxidant activity of 79.9 ± 1.6% and IC_50_ = 414.6 μg/mL. On the contrary, other researchers [32] have described low antioxidant activity. More probably, the antioxidant actions of *M. piperita* may be due to the presence of phenolic constituents in its leaves, including rosmarinic acid and different flavonoids such as rutin, naringin, eriocitrin, luteolin, and hesperidin, which are present in aqueous extracts [33,34], but not in the essential oil. Furthermore, *M. piperita* EO is associated with increased levels of intracellular ROS, which is indicative of an apoptotic process [35] without the loss of the plasma membrane integrity. 

*M. pulegium*, another species of *Mentha*, has been used in traditional medicine to treat numerous illnesses, such as microbial infections and oxidative stress. Kamkar et al. and Cherrat et al. [36,37] have described the lower antioxidative activity of the *M. pulegium* EO with respect to aqueous or methanol extracts. This difference could be due to a lack of diverse antioxidants in the EO. On the contrary, some authors [38,39] have observed a good radical scavenging ability of *M. pulegium* EO compared with ascorbic acid and Trolox. 

Regarding other species of the genus *Mentha* used as antioxidants, *Mentha spicata* EO has been used. In this case, different results were reported; some authors described the antioxidant activity of *M. spicata* EO [40], while others [41] described a weak antioxidant activity. 

*Mentha longifolia* L. (*M. longifolia*) is known as a wild mint named Puneh, and is a fast-growing and perennial herb that creeps along an underground rootstock, which can grow to 1–2 m tall. Eissa et al. [42] revealed that *M. longifolia* EOs possesses the highest scavenging activity against peroxyl radicals. 

*Mentha suaveolens* Ehrh is a communal wild plant that is found near streams, bogs, and humid places. There are different subspecies, each including several varieties. El-Askary et al. [43] reported a potent antioxidant activity in vivo, which was about 96% relative to vitamin E, while Ferreira et al. [44] described the AChE inhibitory capacity as higher than 50% in the essential oil fraction of *M. suaveolens*. The antioxidant capacity in this case is due to piperitone oxide being present at 88% [45]. Other authors have reported no relevant antioxidant activity for this species [46]. Some *Mentha* spp. have not been assessed for antioxidant activity as yet. Table 1 reports antioxidant information about *Mentha* spp. EOs.

## 3. Antifungal Properties 

A worldwide increase in the incidence of fungal infections has been observed, as well as the spread of drug resistance among some species of fungus to different drugs used in medicinal practice [47]. Mycosis is a public health problem, particularly in tropical and subtropical developing countries. *Candida* spp. is the most frequent pathogen in humans and animals. Azole-resistant Candida and Aspergillus species are the pathogens that are responsible for nosocomial or food-borne infections. Although *C. albicans* (*CA*) is the most commonly isolated species, there has also been a significant increase in the frequency of other Candida species, such as *C. parapsilosis*, *C. tropicalis,* and *C. glabrata*. There is emerging evidence from different parts of the world of the increasing resistance of *CA* and non-*CA* species to antifungal agents such as triazoles and echinocandins, which are used in medicinal practice [48]. 

Dermatophytes are a particular group of fungi that infect the keratinized tissues of humans and animals. Infections caused by these fungi are probably the most common cutaneous fungal diseases. Among the dermatophytes, *Trichophyton mentagrophytes* and *Trichophyton rubrum* are cosmopolitan species, and are the most common agents of dermatophytoses. Among other filamentous fungi, the most important is *Microsporum canis*, which is a worldwide distributed zoophilic dermatophyte that is a recurrent cause of ringworm in humans, especially in young people [49]. 

Alternatives to conventional antimicrobial therapy have been studied to resolve this emergence. Many *Mentha* species Eos, such as *M. piperita*, *M. spicata*, *M. longifolia*, *M. pulegium*, *M. cervina,* and *M. suaveolens* have been used to evaluate antifungal activity against different species. Their antifungal activity was evaluated by a variety of different protocols. The minimum inhibitory concentration (MIC) is the lowest concentration of a drug that prevents the visible growth of a microorganism. It can be determined by culturing microorganisms in liquid media. Other studies have also reported the disk diffusion method (DDA), which measures the sensitivity of microorganisms to antibiotics by culturing microorganisms on solid growth media surrounding the sources of the drug.

Therefore, comparing the antifungal activity of different EOs is very difficult, as different methods and ways of expressing concentrations have been used. Sometimes, some authors have reported values of MIC as percent *v*/*v*, μL/mL, or μg/mL. It is more important to highlight that the essential oil weight is about 1 g/mL. When the values of MIC were expressed in μL/mL, a few μL correspond to milligrams of essential oil. The same is true when the MIC values were expressed in percentages. To compare the values of MIC, we have transformed the concentration of all of the results into μg/mL, as reported in Table 2. 

Table 2 shows the antifungal activity of *Mentha* EOs against some human pathogenic fungi. The genus that has been most investigated was *Candida* spp., followed by dermatophytes and *Aspergillus* spp., *M. piperita* and *M. suaveolens* EOs have been studied more than *M. spicata*, *M. longifolia*, *M. pulegium*, and *M. cervinia*. The method that has been more frequently used was a microbroth dilution, rather than disk diffusion. 

It is possible to observe that the values of MIC that are reported are different. Indeed, the values of MIC changed from 0.03 μg/mL, as reported by Oumzil et al. for *M. suaveolens* EO [66], to 7120 μg/mL reported by Mimica-Dukić et al. for *M. piperita* and *M. longifolia* EOs [50].

Mimica-Dukić et al. [50] reported the antifungal activity of three EOs of the *Mentha* species *M. piperita*, *M. longifolia*, and *M. acquatica*. The antifungal activity of *M. piperita* and *M. longifolia* EOs that was evaluated against *CA* were 8 μL/mL, corresponding to about 7120 μg/mL of essential oil. Moreover, the EOs of *M. piperita* and *M. longifolia* were found to be more active than that of *M. aquatica*. *M. piperita* EO against *Trichophyton tonsurans* showed a MIC of 4 μL/mL, corresponding to about 3560 μg/mL. All of the values of MIC expressed in μL/mL are low; in reality, they are the highest.

Tampieri et al. [51] reported values of MIC expressed in ppm (MIC = 500 ppm); when this value was transformed into μg/mL, it corresponded to about 44.5 μg/mL. The antifungal activity of *M. piperita* EO against a strain of CA was evaluated after seven days. 

Other authors [52,53] have shown the antifungal activity of *M. piperita* EO with values of MIC of 225 μg/mL and 256 μg/mL, respectively, for *CA* performed by microbroth dilutions. High values of MIC for *Candida* spp., dermatophytes, and *Aspergillus* spp. were reported by others [54,55,56]. Ibrahim et al. [57] reported antifungal activity against dermatophytes at 890 μg/mL, while recently Ebani et al. [58] did not demonstrate any antifungal effect at the highest dilution against *Aspergillus fumigatus*. In addition, many authors have reported the antifungal activity of *M. spicata* EO, especially against dermatophytes. Both the methods of disk diffusion and microbroth dilution were used. 

Indeed, Soković et al. [54] reported the fungistatic activity of *M. piperita* in the macrodilution and microdilution methods, with values of MIC of 1335 μg/mL to 2670 μg/mL in ethanol and 445 μg/mL to 1335 μg/mL in Tween against *Aspergillus* spp. and dermatophytes. In addition, Sokovic et al. reported the antifungal activity of some main compounds, such as 1–8 cineole, menthol, limonene, and carvone, as shown in Table 3. Better activity was reported for *M. spicata* [54]. Similar results were reported by Khoury et al. [61], with values of MIC of 512 µg/mL against *Tricophitum rubrum,* while high values of MIC were reported by others [62] ranging from 2% *v*/*v* to 3% *v*/*v* and corresponding to about 1780 μg/mL to 2670 μg/mL. Adam et al. [60] reported antifungal activity from 25 mm to 40 mm for disk diffusion and 0.25 μg/mL for microbroth dilutions. *M. spicata* EO possesses greater fungistatic activity than *M. piperita* EO against dermatophytes.

The antifungal activity of *M. longifolia* EO against *Candida* spp. were reported by Ertaş et al. [63]. The activity was evaluated by both methods. The results were interesting, with values of MIC of 3.9 μg/mL.

Similar results were reported for *M. pulegium* EO against *Candida* spp. Indeed, two authors [38,64] reported 19 mm and 16 mm for DDA and MIC values of about 1112.5 μg/mL and 890 μg/mL, respectively. This EO was more active against *Aspergillum* spp. [64]. 

Goncalves et al. [65] showed the antifungal activity of *M. cervina* EO against yeasts, Aspergillus, and dermatophyte strains. All of the species that were tested showed the same high values of MIC, from 1250 μL/mL to 2500 μL/mL and corresponding to about 1112.5 μg/mL to 2225 μg/mL.

Oumzil et al. [66] described the antifungal activity of *M. suaveolens* EO against *CA* and *C. glabrata*. The MIC values, which were expressed in ppm, were in the range of 0.34 to 2.77 ppm, corresponding at about 0.03 μg/mL to 0.24 μg/mL. In addition, Angiolella et al. [67] reported the antifungal activity of the same EO against different dermatophytes such as *Trichophyton mentagrophytes* and *Microsporum canis*. Antimycotic activity was evident for dermatophytes with a range from 0.06% *v*/*v* to 0.125% *v*/*v*, corresponding to about 53.4 25 μg/mL to 111.25 μg/mL for all of the strains of *Trichophyton mentagrophites*, *T. violaceum*, *Microsporum gypseum*, and *M. canis*. El-Askary et al. [43] published the antifungal activity of *M. suaveolens* EO against *CA* and *Aspergillus niger,* with low values of MIC of 4 μg/mL, 5.2 μg/mL, and 6.8 μg/mL, respectively. High values of MIC have been obtained by others [68,69] against *CA,* with MIC values from 390 μg/mL to 780 μg/mL. In addition, Spagnoletti et al. [46] reported the antifungal activity of *M. suaveolens* EO against *Candida* spp., with MIC values ranging from 760 μg/mL to1560 μg/mL. Scazzocchio et al. [70] confirmed the antifungal activity against different strains of *CA* that was sensitive or resistant to fluconazole. This activity has been evaluated by both DDA and MIC. 

The results of the antifungal activity of *Mentha* spp. EOs are highly divergent, and it is possible to observe that *M. suaveolens* EO [66] was more active on *Candida* spp. than *M. longifolia* EO [63], followed by *M. piperita* EO with MIC values of 225 μg/mL to 256 μg/mL [52,53]. *M. spicata* EO was more active on dermatophytes, with values of MIC of 0.25 μg/mL [54], and *Aspergillus* spp. with an MIC value of 313 μg/mL [59], while *M. suaveolens* EO was more active on *Aspergillus* spp. with values of MIC of 6.8 μg/mL [43]. 

## 4. Antibiofilm Properties 

About 65% of infections are caused by microorganisms developing on top of, rather than within, the free-living planktonic state [71,72]. Biofilms are an association of microorganisms that are attached to the surface and encased within a self-produced matrix of exopolymeric substances. Biofilms are constituted by microorganisms with an extraordinary degree of organization. The Candida biofilm consists of a heterogeneous structure that is composed of yeast and hyphae cells surrounded by a self-secreted extracellular matrix. The Candida biofilm frequently shows resistance to conventional antifungal drugs such as amphotericin B and fluconazole. During biofilm formation, an increase in the metabolic activity was associated with an increase in antifungal resistance, which was coincident with biofilm maturation [73]. Biofilm resistance constitutes an important factor in human disease. The biofilm in *Candida* spp. represents an important virulence factor, and its reduction is very important in combating infections. It is alarming that biofilm formation by Candida species may increase resistance to antifungal therapy and protect the microbial cells from the host immune defenses [74]. The possibility of essential oils having potential antibiofilm activity has been studied by many authors. Indeed, Agarwal et al. [75] reported the antibiofilm activity of 30 EOs against *CA*. Four EOs, including eucalyptus, peppermint, ginger grass, and clove, showed 80.87%, 74.16%, 40.46%, and 28.57% biofilm reduction, respectively. Saharkhiz et al. [55] described the inhibition of biofilm formation of *CA* and *C. dubliniensis* with *M. piperita* EOs at concentrations of up to 2000 μg/mL. Stringaro et al. [69] reported that *M. suaveolens* EO was able to reduce biofilm formation by about 80% at concentrations of 780 μg/mL and 1560 μg/mL in sensitive and resistant to fluconazole *CA* strains. Other authors, such as Busato de Feiria et al. [76], observed the antibiofilm activity of three species of *Mentha* (*M. aquatica*, *M. arvensis,* and *M. piperita*), which were able to inhibit biofilm formation and disrupt the mature biofilm of *CA MYA-2876*. These EOs inhibited up to 50% of the biofilm formation at concentrations of 31 μg/mL, 25 μg/mL, and 4000 μg/mL. 

## 5. Cytotoxic Activity

Cancer has been one of the most significant health problems over the last few years. It is a multifactorial condition in which uncontrolled cell growth leads to the formation of solid tumors. Finally, cancer cells invade healthy tissues. This process is known as metastasis.

Scientists are currently studying many synthetic drugs to hit and kill the dividing cells of various tumors [77]. Unfortunately, the drugs that have been used in cancer therapy are not highly selective for tumor cells, but also act against normal cells—mainly against cells with a high reproductive index such as those of the blood. This unfortunately causes well-known side effects such as anemia. Moreover, chemotherapy induces multidrug resistance, which is a phenomenon that demonstrates a broad cross-resistance of cancer cells to various chemotherapeutic agents [78]. 

It has been recognized that various EO components act as multi-target molecules. With the aim of developing novel antitumoral drugs, various EOs have showed high efficacies against human cancer cells and low toxicity to human normal cells.

Many scientific studies have shown that some EO components, such as terpenes, have been found to be effective against a broad range of cancers [79]. An example is geraniol, an acyclic dietary monoterpene that is present primarily in roses and geraniums [80]. 

An *in vivo* study in mice has demonstrated that 5-fluorouracil (20 mg/kg) and geraniol (150 mg/kg) have an important impact against colon cancer. This administration reduces tumor volume by 53%. A low reduction was obtained after a geraniol administration alone, while 5-fluorouracil showed no effect [81].

D-limonene, another monoterpene that is present in many EOs, has shown its efficacy both *in vitro* and *in vivo* against many types of cancers. The scientific results of phase-I clinical trials indicated a partial response in patients with different cancers [82].

Carvacrol and timol, which are phenol monoterpenes present in the EOs of oregano and thyme, have shown an antiproliferative property in the cultured cells of several types of tumors such as lung, liver, and colon cancers [83]. The genus of *Mentha* have also been shown to have many biological activities, and especially anti-tumoral activity. Many studies have demonstrated the effect of *Mentha* species EOs as inhibiting the cell proliferation of numerous tumor cells by acting on mitochondrial dysfunctions, apoptosis induction, and autophagy processes [84]. 

Hajighasem et al. [85] demonstrated that an aqueous extract of *Mentha spicata* leaves has cytotoxic effects on both mouse fibrosarcoma and human leukemia cell lines (Wehi-164), and on human monocytic leukemia (U937) cells, *in vitro*. In addition, Bardaweel et al. [41] reported the antiproliferative activities that are associated with *Mentha spicata* EO against three human cancer cell lines. Sharma et al. [14] reported the activity of extracts of *Mentha* spp. against different human cancer cell lines.

Moderate anticancer activity of *M. longifolia* EO on HepG-2, MCF-7, and A549 human tumor cell lines at a concentration of 100 μg/mL has been reported by Abdel-Hameed et al. [86]. Under oxidative stress conditions, *M. longifolia* oil prevented cell death and inhibited the ROS production that was caused by H_2_O_2_ in a human astrocytoma cell line [42].

**Figure 2:**
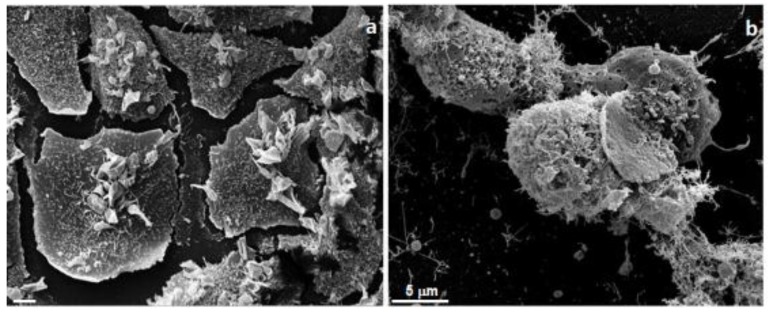
Scanning electron microscopy image of SKBR3 cells (**a**) (5 μm) and incubated with *Mentha suaveolens* EO for 24 h (**b**).

*Mentha suaveolens* plants that were collected in various regions of Morocco contained from 65% to 90% of monoterpenes, oxides, terpenic, and terpenic ketones in their EOs. Recently, Mangone et al. [87] studied the cytotoxicity of *M. suaveolens* EO on the human breast adenocarcinoma cell line SKBR3. Analysis revealed a high percentage of piperitenone oxide, constituting 90% of the EO [45,67]. In this study, the researchers evaluated the cellular metabolism of cancer cells by 3-(4,5-dimethylthiazol-2-yl)-2,5-diphenyltetrazolium bromide (MTT) assay after different treatment with EOs at different times of incubation. Moreover, morphological and ultrastructural studies by electron microscopy analysis were performed to understand the EOs’ cytotoxic action against SKBR3 human cancer cells (Figure 2). Scanning electron micrographs of human breast adenocarcinoma cells treated with *Mentha suaveolens* EO for 24 h at 0.003 mg/mL are shown. Cells appeared damaged on the plasma membrane, with many superficial alterations. 

In the field of biomedical research, both the transmission electron microscope (TEM) and the scanning electron microscope (SEM) play an important role, especially when they are used in association in correlative studies. In fact, they yield complementary morphological and ultrastructural information. 

The standard preparation procedure for electron microscopy requires fixation and dehydration [88,89]. In studies of cancer cells, electron microscopes are instruments that produce a largely magnified image by using electrons instead of light to form an image. TEM and SEM microscopies allow us to study the ultrastructural details of biological samples, and in this case, it is possible to see the changes induced by treatment with several types of agents such as drugs and natural products as EOs. Moreover, it allows us to study the development of any pathological situations, as well as any changes in environmental conditions.

Ultrastructural alterations in the plasma membrane, cytoplasm (swelling, shriveling, vacuolations, leakage) and nucleus are revealed by SEM and TEM [90,91,92]. By electron microscopy analysis, Giordani et al. [93] also demonstrated the plasma membrane alterations of human cancer cells after treatment with an essential oil that was largely composed of monoterpenoids. Accordingly, the antimicrobic effect of monoterpenoids [94] and monoterpenoids’ cytotoxic activities may produce alterations of lipidic fractions and consequently changes in membrane properties. Different EOs or their main components have been shown to induce caspase-dependent apoptosis in human cancer cells. In these cells, EOs can provoke both mitochondrial membrane depolarization [95,96,97] and alterations of membrane fluidity, which become abnormally permeable, resulting in the leakage of numerous molecules. Additionally, as visible by SEM observations, the permeabilization of the outer and inner mitochondrial membranes leads to cell death by apoptosis and necrosis (Figure 2) [98].

## 6. Side Effects and Toxicity 

Numerous publications describe the toxicity of EOs, especially for humans, because despite them being natural substances, whether they are safe is not certain. They are produced by plants in order to protect themselves from parasites and insects, and prevent germination during the winter. For these reasons, they could be toxic for human use, and they should always be used with caution. Toxicity can be local or systemic, which is due to the composition of EOs. At first, the composition of EOs must be verified by gas chromatography because composition and toxicity vary according to different factors such as the seasonal period in which they are collected, the ecotype, the part of the plant from which they are extracted, and the place where the oils are harvested [99,100,101]. The risk assessment of EOs for human consumption is very important, particularly for vulnerable populations such us children and pregnant women. Unfortunately, unlike with typical regulated drugs, rigorous scientific tests have not been established for the safety of EOs. Toxicology studies in mice have revealed that *Mentha pulegium* L. is hepatotoxic due to the ketone function of its volatile component, pulegone [99]. For this reason, the maximum dose of this terpene in mint candies is 250 ppm [99], while in cosmetic products, it should not exceed 1% [102], because it may cause irritation to the skin and mucous membranes [102]. 

*Mentha* spp. EOs are widely used for clinical purposes, mostly as an ingredient in creams for the treatment of musculoskeletal pains and as an analgesic for arthritis due to its property of reducing painful muscle spasms. Moreover, peppermint oil (*Mentha piperita* L.) is used for soothing headaches, reducing spasms of the intestinal tract, and for symptoms of indigestion. It has a good antispasmodic action that relaxes the gastrointestinal tract [12,103,104], and is also good for neuralgia. 

Furthermore, toxicity can depend on the route of administration, and on the health conditions of the individual using the EOs (damaged skin, allergy). Additionally, it depends on the additives added to the oil. Unfortunately, there are many side effects related to *Mentha* spp. EOs, such as nausea, allergic reactions, and flushing. These effects are due to the pharmacokinetic interactions that may occur when EOs are taken together with conventional drugs. In particular, peppermint can interfere with cytochrome P450; this cytochrome is fundamental in drugs and chemical metabolism [104].

For this reason, anyone who produces the EOs and in particular *Mentha* spp. EOs should refer to the guidelines published, for example, by the European Federation of Essential Oils (EFEO) or by the International Fragrance Association (IFRA) for the correct characterization of the essential oil.

## 7. Conclusions

*Mentha* spp. EO has been used historically for several health conditions and exists in many countries as a medicinal product with authorization. *Mentha* spp. EOs are employed in many fields such as flavoring, medicine, cosmetology, and toiletries, but could represent an excellent opportunity for integrative medicine to be used in combination with therapy for human health or could be useful for extracting molecules to be used to formulate new drugs.

In this current review, only some biological properties of *Mentha* spp. EOs, such as their antioxidant, antifungal, antibiofilm, and cytotoxic properties, are considered. It is very difficult to compare studies on these factors, because there are many differences both in the extraction of the essential oil and in the methods that are used to study its biological activities. 

Many further studies are needed to understand the mechanism of action and to establish the safety and non-toxicity of these EOs.

## Figures and Tables

**Table 1:** Radical scavenging activity of *Mentha* essential oils (EOs). ABTS: 2,2’-azino-bis 3-ethylbenzthiazoline-6-sulphonic acid, DPPH: 2,2-diphenyl-1-picrylhydrazyl.

EOs	DPPH Activity	ABTS Activity	References
***M. piperita***	860 μg/mL	-	[28]
	57.9 ± 1.34%	80.6 ± 1.45%	[29]
	600 μg/mL	-	[30]
	540 μg/mL	-	[31]
	11.289 ± 0.514 μg/g	0.154 ± 0.006 mmol/g	[32]
***M. pulegium***	14736 ± 156 μg/mL	-	[36]
	30.38 ± 0.8%	-	[37]
	69.60 μg/mL	-	[38]
	321.41 ± 2.53 μg/mL	-	[39]
***M. spicata***	3 μg/mL	-	[40]
	3450 ± 172.5 μg/mL	40.2 ± 0.2 μg/mL	[41]
***M. longifolia***	57.4 μg/mL	-	[42]
***M. suaveolens***	31 μg/mL	-	[43]
	52.4 ± 2.5%	-	[44]

**Table 2:** Antifungal activity of *Mentha* spp. EOs against human pathogenic fungi.

*Mentha* spp.	*Candida* spp.		Dermathophytes		*Aspergillus* spp.		References
	MIC ^1^ μg/mL	DDA ^2^ mm	MIC μg/mL	DDA mm	MIC μg/mL	DDA mm	
***M. piperita***	7120	-	3560	-	-	-	[50]
	44.5	-	-	-	-	-	[51]
	445	90	-		-		[16]
	225	-		-	-	-	[52]
	256	-	-	-	-	-	[53]
	-	-	−1335–2670	-	1335–2670	-	[54]
	1068–3560	-	-	-	−445–3560	-	[55]
	890	-	-	-	-	-	[56]
	-	-	890-	-	-	-	[57]
	-	-	-	-	No activity	-	[58]
	625	-	-	-	625	-	[59]
***M. spicata***	-	-	0.25	25 to 40	-	-	[60]
	-	-	890–2225	-	890–2225	-	[54]
	-	-	512	-	-	-	[61]
	-	-	1780–2670	-	-	-	[62]
	625	-	-	-	313	-	[59]
***M. longifolia***	7120	-	-	-	-	-	[50]
	3.9	2.5	-	-	-	-	[63]
***M. pulegium***	890	16	-	-	222.5	10	[64]
	1112.5	19	-	-	-	-	[38]
***M. cervinia***	1112.5 to 2225	-	1112.5–2225	-	1112.5–2225	-	[65]
***M. suaveolens***	0.03 to 0.24	-	-	-	-	-	[66]
	-	-	53.4–111.25	-	-	-	[67]
	4	-	-	-	6.8	-	[43]
	390 to 780	-	-	-	-	-	[68]
	780	-	-	-	-	-	[69]
	760 to 1560	-	-	-	-	-	[46]
	780	17 to 35	-	-	-	-	[70]

Antifungal activity of *Mentha* spp. EOs. ^1^ MIC: minimum inhibitory concentration; ^2^ DDA: Disk Diffusion Agar performed with 10 μL of essential oil.

**Table 3:** Antifungal activity of main compounds of *Mentha* spp. Eos.

Compound	MIC (μg/mL)
1,8 cineole	2760 to 7360
Menthol	220 to 1320
Limonene	500 to 1000
Carvone	240 to 960
